# Transcultural adaptation of the virtual patient integration rating scale: a factor analysis

**DOI:** 10.1186/s12909-024-06571-z

**Published:** 2025-03-18

**Authors:** Isaza-Restrepo Andrés, Buitrago-Ricaurte Natalia, Bermúdez-Hernández Pablo, Ariza-Salamanca Daniel, Ibañez-Pinilla Milciades, Vergel John

**Affiliations:** 1 School of Medicine and Health Sciences, Medical and Health Sciences Education Research Group, Universidad del Rosario, Bogotá, Colombia; 2 School of Medicine and Health Sciences, Medical and Health Sciences Education Research Group, Cannon Research Group, Universidad del Rosario, Bogotá, Colombia

**Keywords:** Medical education, Teaching, Clinical reasoning, Medical students, Avatar, Curriculum

## Abstract

**Background:**

Using Virtual Patients (VPs) in medical education has gained popularity, especially during the SARS-CoV-2 pandemic, which restricted traditional clinical training. VPs provide a learning platform for students to refine their clinical reasoning and decision-making skills in a risk-free environment. Although the educational benefits of VPs are well known, there is still a need for validated tools to assess student perceptions, which are key to optimizing learning outcomes. The Virtual Patient Integration Rating Scale (VPIRS) has made a valuable contribution in this regard, having been established in English-speaking contexts, but its applicability in Ibero-American countries remains poorly explored. This study aimed to fill this gap by transculturally validating VPIRS for Spanish-speaking medical education environments, ensuring it reflects cultural nuances.

**Methods:**

We conducted a two-phase transcultural validation of the VPIRS on medical students at the Universidad del Rosario. First, we translated and culturally adapted the VPIRS, using the modified Delphi method for face validation. Second, we assessed the construct validity, internal consistency, reproducibility, and reliability of the scale through a test-retest approach with 153 participants, using descriptive statistics, factor analysis, and reliability testing in SPSS.

**Results:**

The VPIRS was successfully adapted and validated for transcultural use in Spanish (VPIRS-E). The exploratory and factor analyses maintained the original scale’s four-dimensional structure, explaining 61.8% of the total variance, with an overall Cronbach’s alpha of 0.826. Test-retest reliability demonstrated robust intraclass correlation coefficients ranging from 0.8 to 0.9.

**Conclusion:**

The VPIRS-E is a reliable and valid instrument that has maintained the structural integrity of the original scale and has demonstrated strong internal consistency across all of its domains. These results demonstrate the suitability of the VPIRS-E to assess medical students’ perceptions of the use of VPs in Spanish-speaking learning contexts. The successful validation of this instrument also opens avenues for expanded international comparative studies, allowing fora deeper understanding of the effective integration of VPs in different medical education curricula.

**Supplementary Information:**

The online version contains supplementary material available at 10.1186/s12909-024-06571-z.

## Background

Virtual patients (VPs) have been used in medical education to enhance students’ clinical skills, while ensuring that no real patients are harmed [1]. This approach facilitates early exposure to professional demands and integration of essential concepts that strengthen students’ critical reasoning [2]. Numerous studies have shown that integrating VPs into the medical curriculum improves students’ diagnostic, treatment, and communication skills [3–6]. Furthermore, due to the SARS-CoV-2 pandemic’s limitations on students’ hospital and primary care rotations [7, 8] there has been a considerable increase in the use of VPs for instructional purposes. Accordingly, the perception of VPs by students has become a relevant research topic.

Despite the increased interest in researching VPs in medical education, the current academic debate has primarily focused on the development and implementation of instructional designs that integrate this technology. The research on VPs shows a wide variance in context and methodology, making direct comparisons difficult and obscuring a clear understanding of their educational value [9]. Some studies have evaluatedthe effectiveness of VPs in enhancing students’ clinical skills compared to real patients [6, 10, 11] with most of these studies using qualitative or mixed-method approaches [12, 13]. However a challenge in this area of research is the limited literature available on tools that assess students’ perceptions of integrating VP into their curriculum. While some studies have explored this issue, their focus is on different disciplines, contexts, and purposes than those of our research [14, 15].

The Virtual Patient Integration Rating Scale (VPIRS) was specifically designed to measure medical students’ perceptions of VPs, focusing on their role in enhancing clinical reasoning and semiology training as an additional teaching and learning strategy. The VPIRS consists of 25 Likert-scale items in four domains: knowledge acquisition, learning facilitation, non-authentic teaching, and learning disabilities [14–18].

Another distinctive feature of VPIRS is its ability to independently assess clinical skills such as history taking, physical examination, and differential diagnosis, instead of combining them into broader categories. In addition, VPIRS is particularly well suited to educational environments that emphasize autonomous learning, as it allows students to interact with virtual patients in an autoregulated manner without needing constant supervision by a facilitator.

Although the VPIRS was originally developed and traduced in Slovenian [18], it has been used in English-speaking contexts. However, to the best of our knowledge, there is no validated Spanish version available for Ibero-American countries. This gap creates uncertainty about students’ perceptions of VPs in these contexts and hinders global comparative analyses. A directly translated VPIRS without thorough transcultural validation for the specific needs of Spanish-speaking medical students may yield unreliable results. Thus, the aim of this study was to translate and transculturally validate the VPIRS to accurately assess Spanish-speaking medical students’ perceptions of VPs use.

## Methods

### Study settings and participants

This adaptation and transcultural study involved first through sixth year medical students at the Universidad del Rosario. Participants were selected from those who had completed at least one course using VPs. Located in Bogota, Colombia, our university enrolls approximately 280 medical students per year. The six-year medical curriculum emphasizes the integration of basic/biomedical, clinical, population health, and sociohumanistic sciences. This integration revolves around the Problem-Based Learning (PBL) methodology, complemented by laboratories, lectures, and semiology training in real clinical scenarios starting in the first year.

As part of this curriculum, VPs are incorporated through the i-Human^®^ platform by Kaplan. The platform simulates patient encounters through avatars and detailed animations illustrating physiological, pathological, histological, and anatomical systems. Students are required to engage with these cases in English, with language proficiency certifications required throughout the program. Students interact with a curated set of cases selected and standardized by clinical experts as part of the university’s e-Clinic: Transcurricular Virtual Clinical Strategy. The learning experience has two main components: first, students work independently or in small groups to analyze the VP cases, focusing on history taking and physical examination. Then they present their findings in simulated medical rounds, where they receive formative feedback from facilitators. Second, students participate in real-time case evaluations under supervision, where they apply their knowledge to diagnose and manage VPs and generate analytical reports. This experience is designed to strengthen both clinical reasoning and semiological skills, and to prepare students for future interactions with real patients in clinical settings.

For the survey, we used non-probabilistic sampling to select our participants based on voluntary students’ participation and previous exposure to VPs. And following the best practices recommendations [16], we determined a sample size of 150 students. This calculation was based on the criterion of having at least 5 students per scale item. To reduce the risk of data loss or attrition, we included an additional 20% buffer, following the approach of similar studies [17].

### The scale

We did a literature review to identify existing tools suitable for assessing student perceptions of VPs use. In PubMed, the search criteria was: ‘Perception*[Title/Abstract] AND ((“virtual patients“[Title/Abstract] AND “perception“[Other Term] AND “scale“[Other Term] AND “students“[Other Term]) OR “virtual patients“[Other Term]) AND “students“[Other Term].’ In Google Scholar, the search terms used were ‘(virtual patient scale perception “medical students”).’ This process led us to the Virtual Patients Integration Rating Scale (VPIRS), a tool initially developed in a Slovenian-speaking context and translated to English. The VPIRS, recognized for itspsychometric properties, assesses medical students’ perceptions of virtual patient-based learning. This instrument has 25 items and its responses are recorded on a 5-point Likert scale. Ratings range from 1, signifying strong disagreement, to 5, indicating strong agreement. The scale is divided into four distinct domains: Knowledge Acquisition and Retention, Learning Facilitation, Perceptions of Inauthentic Teaching, and Learning Disadvantages. Given its established validity and relevance to our pedagogical aims, we obtained the author’s authorization to translate and transculturally adapt the VPIRS for our study [18].

### Translation, adaptation, and validation process

Our methodology comprised two phases. The first phase, drawing from Mohamad-Isa et al.’s method, involved translation and face validation [19, 20]. The second phase centered on assessing content, and construct validity, as well as internal scale consistency and reliability [16].

#### First phase

The VPIRS was translated into Spanish by two certified bilingual translators. Our research team reviewed and merged these translations into a single version. This Spanish version was then back-translated into English by two bilingual experts in medical education. Using the modified Delphi method, the research team refined this back-translated version to a preliminary state, ensuring it retained the original number of items and the five-point response scale (ranging from ‘strongly disagree’ to ‘strongly agree’).

**Fig. 1:**
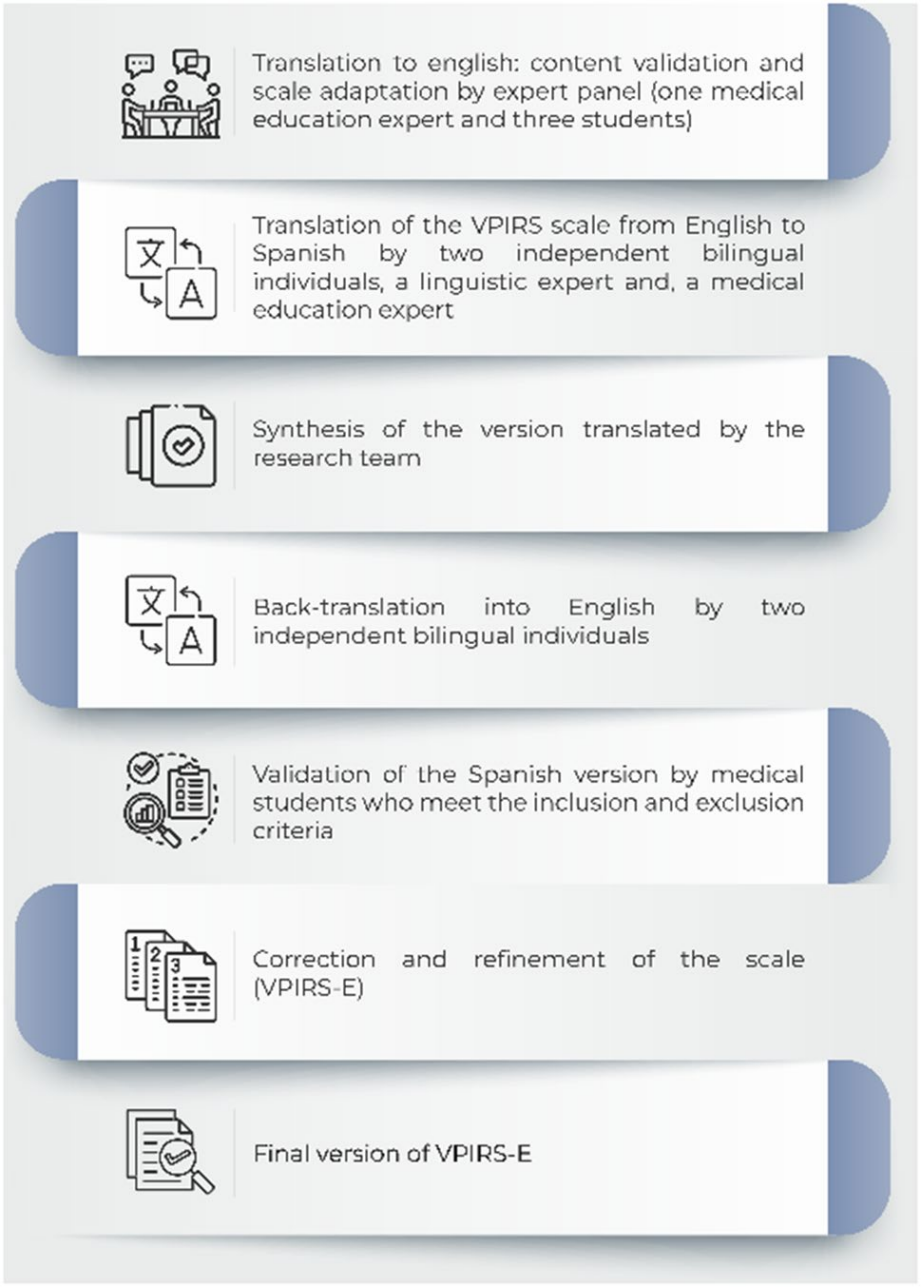
Protocol for the cross-cultural validation of the scale

We then tested this preliminary version for face validity on ten medical students of varying years, who had experience with VPs. In this method, students independently solve pre-set virtual patient cases. This trial helped evaluate the scale’s face validity and identify any idiomatic or cultural nuances (see Fig. 1). We assessed the scale’s acceptability through the discrimination index [21].

#### Second phase

**Fig. 2:**
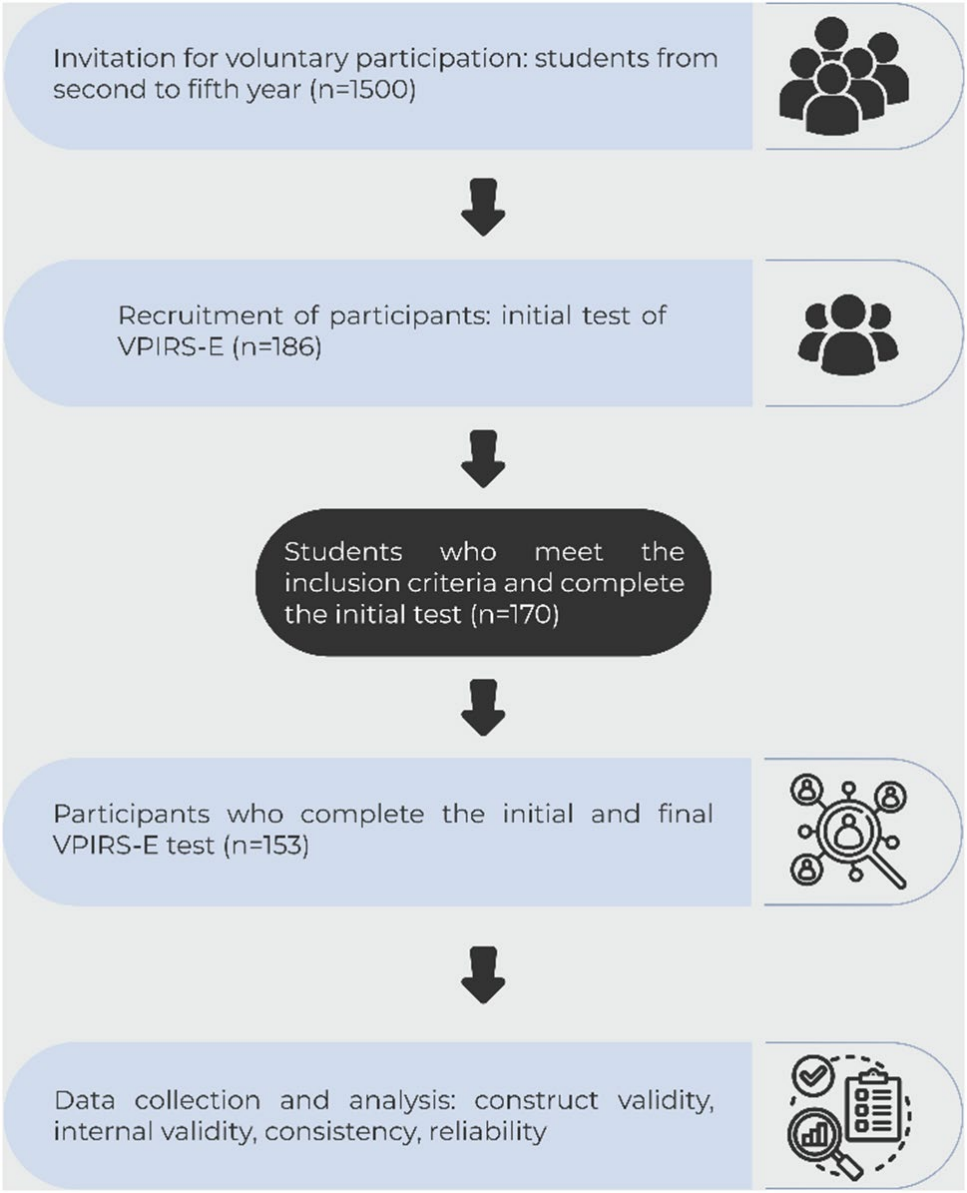
Established protocol to evaluate the scale’s internal consistency, and reliability

We administered the revised version of the scale to medical students who had previously engaged with VPs. Our objectives were to establish construct validity, internal consistency, reproducibility, and reliability of the scale (see Fig. 2). We obtained prior informed consent from the participants and employed a test-retest methodology for this purpose. The scale was administered under the supervision of two medical education experts of the research team.

To ensure the reproducibility of our findings, we scheduled the retest after a one-month interval, coinciding with the students’ vacation period. This timing was strategic, as it minimized the likelihood of students’ further exposure to VPs between tests, thereby reducing potential bias in their responses.

### Data collection and analysis

The study began by extending invitations for voluntary participation to all of our nearly 1,500 students. Detailed explanations about the study’s purpose and methodology were provided, emphasizing the students’ rights to voluntary participation and assuring confidentiality. Informed consent was obtained and meticulously documented for those who agreed to participate. Questionnaires were administered in an online format from July to August 2022.

The reproducibility of the data was assessed using the Wilcoxon signed-rank test, which was chosen due to the nonparametric nature of the paired sample data and the fact that the distribution of the scores did not meet the assumptions of normality, as confirmed by the skewness and kurtosis coefficients, as well as the Kolmogorov-Smirnov and Shapiro-Wilk tests. While correlation-based methods (e.g., Pearson’s or Spearman’s correlation) are typically used for test-retest reliability, given the distributional characteristics of the data, the Wilcoxon test was more appropriate for capturing both the magnitude and direction of changes in individual scores over time. Then, exploratory factor analysis was conducted to examine the scale’s factor structure. This step was essential to understand the underlying dimensions of the scale. Reliability analysis, including the calculation of Cronbach’s alpha, was performed to determine the internal consistency of the items.

To further validate the factor structure identified in the exploratory and confirmatory analysis, factor analysis was undertaken. This involved applying direct oblimin rotation and the maximum likelihood method. We also calculated the Average Variance Extracted (AVE) to establish convergent validity, and correlation coefficients were computed to assess discriminant validity between the factors of the instrument. All statistical analyses were conducted using SPSS (SPSS Statistics for Windows, Version 17.0, Chicago: SPSS Inc). In our analysis, we adhered to a standard of statistical significance set at a p-value of < 0.05.

## Results

This section reports the results across two distinct phases. The first phase involved translating, adapting, and transculturally validating the scale. The second phase focused on evaluating the scale’s construct validity, internal consistency, reproducibility and reliability. Furthermore, this section describes the challenges encountered during the translation phase.

### Translation, adaptation, and transcultural validation of the scale

In our efforts to refine and adapt the instrument for a cultural context different from that of the original English version, we embarked on a meticulous analysis of its Spanish translation. During these sessions, five experts in medical education discussed the nuances of language and meaning. We determined and included only the relevant and representative items. We encountered a challenge in the domain of ‘Acquiring and Maintaining Knowledge.’ The wording of item 5 sparked considerable debate. In the context of virtual patient cases, the term ‘examinations’ in Spanish appeared to be ambiguous, blurring the distinction between a physical examination and a comprehensive case analysis. Similarly, the reference in item 8 to ‘important clinical presentations’ in Spanish was open to interpretation, leaving us uncertain whether it pertained to the techniques used to present patients or to the set of symptoms central to a diagnosis. We opted to specify ‘examinations’ as referring explicitly to physical examinations, since other items within the domain assess aspects related to case analysis.

In the domain of ‘Facilitation of learning’, challenges were also identified in the Spanish versions of item 16, ‘Virtual patient study obligations can be carried out concurrently’, and in item 18, ‘Virtual patients are a useful way of continued medical education (excluding thought courses)’. We noticed that the term “thought” in item 18 might be a typographical error from the original article, and the correct term should probably be “taught” (enseñado). Additionally, we encountered ambiguities in the interpretation of terms such as ‘study obligations’ and ‘continuing medical education’. Our solution to these linguistic challenges was a thorough face validation process. Ten participants answered the scale and an acceptable discrimination index was considered above 90%. This process highlighted the difficulties that participants encountered, for example, with the concept of ‘academic obligations’ in item 16. As a result, we decided to list these obligations explicitly for absolute clarity. Similarly, the Spanish version of ‘continuing medical education’ in item 18 was unfamiliar to many, so we included a concise definition.

The challenges extended beyond these items. In Domain 3, ‘Inauthentic Teaching,’ Item 19 posed a unique dilemma. The Spanish terms elicited a range of interpretations, from ‘not real education’ to ‘education using overly artificial methods.’ To prevent misinterpretation, we refined these terms and produced a final Spanish version of the instrument that was more than just a translation; it served as a cultural bridge. This version included clear instructions and carefully defined terms such as ‘academic obligations,’ ‘continuing medical education,’ and ‘inauthentic teaching,’ ensuring that each question was aligned with our audience’s linguistic and cultural ethos (detailed in Table 1 of the supplementary material 1). These modifications were critical, not mere edits, but instrumental in enhancing students’ understanding, underscoring the important aspects of each question, and embedding culturally relevant language into the instrument.

**Table 1:** Final Spanish version of *virtual patient integration rating scale* (VPIRS-E)

Ítem	Dimensión 1: Adquirir y retener el conocimiento
1	Aprender a través de pacientes virtuales me ha ofrecido mucho conocimiento nuevo.
2	Los pacientes virtuales me han ofrecido la oportunidad de profundizar en el conocimiento que he adquirido hasta ahora
3	Los diferentes métodos de enseñanza que se han utilizado con los pacientes virtuales (fotos, texto) han hecho que aprender sea más fácil.
4	Aprender con pacientes virtuales me ha facilitado una mejor comprensión del cuidado integral del paciente.
5	El examen físico de los pacientes virtuales me permite retener más fácil el conocimiento
6	El requisito de proponer un diagnóstico diferencial me ayudará a obtener la habilidad de razonamiento clínico y priorizar el diagnóstico más probable cuando encuentre a un paciente con síntomas similares en la sala de espera.
7	Tengo un mejor conocimiento de cómo tratar pacientes con síntomas similares a los estudiados en los pacientes virtuales.
8	He aprendido más acerca de las presentaciones clínicas de las enfermedades relevantes para el trabajo práctico.
9	Los casos de los pacientes virtuales son un componente curricular apropiado.
10	He aprendido más sobre el abordaje general del paciente mediante los pacientes virtuales.
11	El programa de pacientes virtuales me ha ayudado a desarrollar habilidades para una anamnesis dirigida.
12	El programa de pacientes virtuales me ha ayudado a desarrollar habilidades para un examen físico dirigido.
13	Los pacientes virtuales me han ayudado a desarrollar mi pensamiento para ampliar los diagnósticos diferenciales.
14	He aprendido cómo actuar con pacientes en escenarios específicos.
**Dimensión 2: Gestión del aprendizaje**	
15	Las otras obligaciones académicas dejan tiempo suficiente para mi estudio independiente de los pacientes virtuales.
16	Las diferentes obligaciones académicas relacionadas con el paciente virtual se pueden llevar a cabo al mismo tiempo. (Obligaciones académicas entendidas como: construcción de historia clínica, revisión de literatura en torno al caso, preparación de la presentación del caso e identificación de hallazgos claves.)
17	Es una ventaja tener la oportunidad de decidir por mi cuenta cuándo completar mis tareas de estudio con mi paciente virtual.
18	Los pacientes virtuales son una herramienta útil para la educación médica continuada. (Entiéndase educación médica continuada como actividades diferentes a los cursos obligatorios)
**Dimensión 3: Enseñanza inauténtica (Enseñanza auténtica entendida como la estrategia que conecta a las asignaturas con el mundo real)**	
19	La educación con pacientes virtuales no es el aprendizaje teórico auténtico.
20	Tengo dudas de que el tratamiento del paciente virtual se corresponda con el del paciente real
21	En muchas ocasiones, los casos de pacientes virtuales son demasiado ideales.
**Dimensión 4: Desventajas para el aprendizaje**	
22	Los casos fueron muy exigentes para mi nivel de conocimiento.
23	Los casos de los pacientes virtuales ocuparon demasiado de mi tiempo.
24	No poder discutir los casos de pacientes virtuales con mi profesor era una desventaja.
25	Entendí poco los casos de los pacientes virtuales en inglés

### Construct validity, internal consistence, reliability and reproducibility

In our study, 186 medical students answered the first test, and 153 completed the questionnaire. The participant group had an average age of 22 years (SD 5 years), predominantly female (73.8%). Most students (81.7%) were in their second to sixth academic years, with the remainder (18.3%) in the seventh year or higher. A comprehensive analysis yielded 3825 complete responses, with no data missing.

### Construct validity: factor analysis

The overall mean response was 3.66, with a standard deviation of 0.98. All items demonstrated sufficient variance, qualifying them for inclusion in the factor analysis (refer to Table 2). The data’s suitability for factor analysis was confirmed through the Kaiser-Meyer-Olkin (KMO) and Bartlett tests. The KMO criterion yielded a value of 0.913 (ideal range 0.5 to 1), and Bartlett’s test of sphericity showed statistical significance (*p* < 0.0001; 300gl; Chi-square 21.256), rejecting the null hypothesis of the presence of orthogonal variability. These results indicated favorable conditions for factor analysis and the identification of factors underlying the correlation matrix of the evaluated items.

### Exploratory factor analysis

**Fig. 3:**
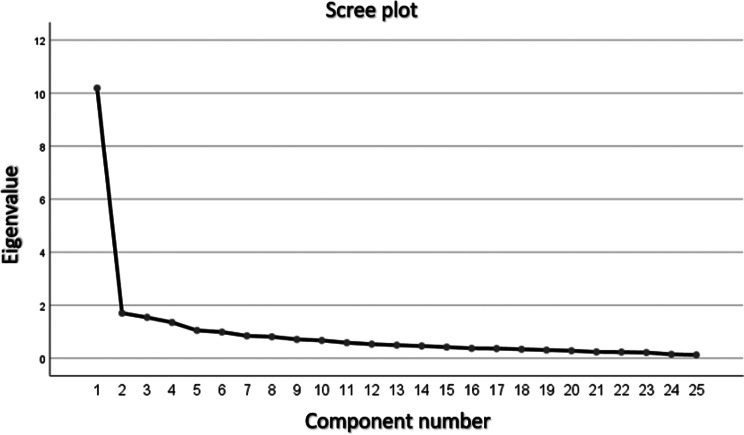
Scree plot, preliminary analysis for the identification of possible components

Our exploratory factor analysis, visualized in the scree plot (Fig. 3), suggested a model with four distinct factors, each with an eigenvalue exceeding 1. This model was chosen as it explained a total variance of 61.8%, a substantial proportion that indicates that these factors are capturing the essence of the data.

In the analysis of commonalities, which reveals how much of each variable’s variance is explained by the factors, we observed a range in values. The highest commonality was 0.702, indicating substantial shared variance for that variable, while the lowest was 0.538, suggesting a more moderate level of shared variance (details can be found in Table 2). This analysis sheds light on the degree to which each variable is related to the factors identified, offering a deeper insight into the data’s underlying structure.

**Table 2:** Descriptive statistics of evaluated items analyzed by four factors

Item	*n*	Mean	SD	Variance percentage (%)	Total variance explained (%)	Communalities
1	153	4.12	0.814	40.106	40.106	0.702
2	153	4.27	0.780	10.158	50.264	0.660
3	153	3.92	0.984	6.095	56.359	0.598
4	153	3.78	1.090	5.508	61.866	0.654
5	153	3.37	1.223	3.803	65.669	0.649
6	153	4.50	0.619	3.200	68.870	0.569
7	153	4.07	0.879	2.925	71.795	0.569
8	153	4.15	0.849	2.871	74.665	0.658
9	153	4.24	0.918	2.584	77.249	0.661
10	153	3.85	1.012	2.428	79.677	0.607
11	153	4.29	0.808	2.282	81.959	0.516
12	153	3.62	1.187	2.103	84.062	0.616
13	153	4.27	0.858	1.877	85.939	0.605
14	153	3.64	1.055	1.748	87.687	0.621
15	153	3.22	1.090	1.612	89.299	0.652
16	153	3.65	0.997	1.499	90.798	0.727
17	153	4.46	0.787	1.400	92.198	0.365
18	153	4.21	0.908	1.325	93.524	0.648
19	153	2.98	1.085	1.233	94.757	0.612
20	153	3.26	1.202	1.082	95.839	0.673
21	153	3.54	1.007	0.997	96.836	0.640
22	153	2.18	0.954	0.912	97.748	0.667
23	153	2.75	1.096	0.811	98.559	0.668
24	153	3.40	1.205	0.800	99.359	0.538
25	153	1.87	1.157	0.641	100.000	0.594

### Definitive factor analysis: internal consistency

We conducted a principal components analysis with Varimax rotation and Kaiser normalization, using four components. This approach explained a total of 61.8% of the variance in the data. The variance distribution among the identified factors is detailed below:

- Factor 1 accounted for 33.15% of the total variance, encompassing items 1 to 14.
- Factor 2 contributed 11.15% to the total variance, covering items 15 to 18.
- Factor 3 was responsible for 9.2% of the total variance, including items 19 to 21.
- Factor 4 represented 8.3% of the total variance, with items 22 to 25.

This distribution clarifies each factor’s contribution to the overall variance and the specific items associated with each.

In our principal component’s analysis using Varimax rotation and Kaiser normalization with four components, we clarified the variance distribution within our data, which totaled 61.8%. Specifically, Factor 1, comprising items 1 to 14, accounted for 33.15% of this variance. Factor 2, including items 15 to 18, contributed 11.15%, while Factor 3, with items 19 to 21, was responsible for 9.2% of the variance. Finally, Factor 4, encompassing items 22 to 25, represented 8.3%. This breakdown effectively delineates the contribution of each factor to the overall variance, along with the specific items grouped under each factor.

In the rotated component matrix, the four components effectively grouped the 25 items, showing satisfactory factor loadings (refer to Table 3 for details). Items 17 and 18, originally intended to measure learning management within Dimension 2, exhibited a stronger association with Dimension 1, focusing on knowledge acquisition and retention. This suggests that aspects of learning management are significantly influenced by the dimension of knowledge acquisition and retention, reflecting the intertwined nature of these educational constructs.

The four components in the rotated component matrix effectively group the 25 items, demonstrating satisfactory factor loadings (see Table 3). Notably, two items (17: “It is an advantage to have the opportunity to decide on my own when to complete my study assignments with my virtual patient” and 18: “Virtual patients are a useful tool for continuing medical education”) from dimension 2, which assesses learning management, are more strongly associated with dimension 1, assessing knowledge acquisition and retention. This finding suggests that these two items, originally designed to measure learning management, are more influenced and explained by the knowledge acquisition and retention dimension. It is plausible that learning management, being a complex and hierarchical phenomenon, involves knowledge acquisition. The theoretical constructs underlying these dimensions may also intersect, leading to the observed overlap and association.

For dimension three, including items 19 through 21 that measure inauthentic teaching, we observed that these items may be influenced by factors related to learning management, as indicated by their loading on factor 2. This connection is further reinforced by the behavior and loadings of items 15 and 16, which relate to time management and concurrency of academic obligations in the third dimension. These factors seem to influence students’ perceptions of the authenticity of the learning process. In addition, perceptions of inauthentic teaching may also influence how students perceive their ability to manage time and balance academic obligations, suggesting a bidirectional relationship between the perceived authenticity of the learning experience and the management of academic tasks in VP-based learning.

Furthermore, Item 24, which falls under the dimension 4 assessing learning disadvantages, demonstrated a closer alignment with the learning management component. This finding indicates that students perceive the absence of teacher interaction in virtual patient cases as a significant disadvantage, underscoring the crucial role of facilitator involvement in effective learning experiences with virtual patients.

**Table 3:** Principal component analysis of the four identified factors

Item	Component			
	1	2	3	4
1	0.774	− 0.272	0.148	− 0.086
2	0.750	− 0.189	0.150	− 0.197
3	0.722	− 0.185	0.193	0.067
4	0.744	− 0.219	0.188	0.132
5	0.612	− 0.386	0.277	0.222
6	0.680	0.103	0.030	− 0.308
7	0.667	− 0.099	0.338	− 0.021
8	0.771	0.011	0.202	− 0.155
9	0.768	− 0.165	0.193	− 0.079
10	0.742	− 0.191	0.012	0.138
11	0.677	− 0.169	0.021	− 0.166
12	0.683	− 0.303	0.157	0.183
13	0.761	− 0.027	0.076	− 0.136
14	0.603	− 0.215	0.393	0.238
15	0.313	− 0.019	0.744	− 0.005
16	0.283	0.135	0.793	0.024
17	0.476	0.152	0.130	− 0.313
18	0.751	− 0.140	0.240	− 0.083
19	− 0.209	0.715	0.087	0.223
20	− 0.241	0.767	− 0.076	0.140
21	− 0.146	0.779	− 0.035	0.106
22	0.050	0.338	− 0.045	0.741
23	− 0.258	0.353	− 0.454	0.520
24	− 0.020	0.419	− 0.512	0.316
25	− 0.082	0.119	0.045	0.756

### Reliability

Encouraging results were found in the reliability assessment of the scale. The overall Cronbach’s alpha for all domains was 0.826, indicating strong reliability. Breaking this down further, Domain 1, which includes items 1–14, showed an impressive alpha of 0.938. Domain 2, covering items 15–18, had an alpha of 0.705. Domain 3, with items 19–21, yielded an alpha of 0.787, and Domain 4, encompassing items 22–25, also recorded an alpha of 0.705. These values attest to the very favorable reliability of each domain within our scale.

A Cronbach’s alpha of 0.826 was found for all the domains. A very favorable reliability is established for this scale and each of the domains evaluated:

- Domain 1: items 1–14, Cronbach’s alpha: 0.938.
- Domain 2: items 15–18, Cronbach’s Alpha: 0.705.
- Domain 3: items 19–21, Cronbach’s alpha: 0.787.
- Domain 4: items 22–25, Cronbach’s alpha: 0.705.

### Reproducibility

We evaluated the instrument’s reproducibility by administering it at two distinct points in time. The Wilcoxon signed-rank test revealed statistically significant differences in the scores for 7 of the 25 items evaluated. Specifically, notable differences were observed in the scores for Item 2 (Z= -3.078; negative range; *p* = 0.002), Item 4 (Z= -3.655; negative range; *p* < 0.001), Item 9 (Z= -2.481; negative range; *p* = 0.013), Item 12 (Z= -2.674; negative range; *p* = 0.008), Item 17 (Z= -3.988; negative range; *p* < 0.001), Item 23 (Z= -2.881; negative range; *p* = 0.004), and Item 24 (Z= -2.999; negative range; *p* = 0.003). The most significant variations in the reproducibility analysis were observed in the first domain (Z= -3.571, *p* < 0.005) and the fourth domain (Z= -2.59, *p* = 0.009). A significant difference was also noted between the first and second applications of the tool overall (Z= -3.57, *p* < 0.005).

## Discussion

This study aimed to transculturally validate the VPIRS for assessing medical students’ perceptions regarding the integration of virtual patients into the curriculum. Our results confirm the Spanish version (VPIRS-E) as both reliable and effective in measuring these perceptions, preserving the original scale’s structure of four dimensions and 25 items. These findings ensure the VPIRS-E accurately reflects the intended constructs, affirming its applicability in Spanish-speaking educational settings.

We also found that certain terms of the scale translated into Spanish were challenging to understand, such as ‘study obligations’ (Item 16), ‘continued medical education’ (Item 18), and ‘inauthentic learning’ (Item 19). The literal translation of these terms resulted in different connotations within our cultural context compared to their original meanings in English. For instance, the term ‘inauthentic learning’ may be interpreted as artificial, synthetic, or rigid. To avoid confusion and enhance understanding, we added descriptive notes to these concepts in the survey form next to the relevant items. We recognized that students were not fully familiar with these definitions and often relied on an intuitive understanding. This careful face validation process highlighted the impact of polysemy on the interpretation of potentially unfamiliar or ambiguous concepts and underscored the critical need to account for linguistic and cultural specificities in the educational context to maintain the scale’s measurement validity. Other studies also emphasize the importance of transcultural translation and adaptation of scales to ensure their reliability, which aligns with our findings [22, 23].

Likewise, we found significant variability within the first and fourth domains (items: 2, 4, 9, 12, 17, 23, 24). This variability may stem from the first domain’s large number of items and the fourth domain’s focus on VPs’ learning disadvantages. These factors likely make these domains more prone to fluctuations over time. In this study, the scale’s second application followed a period without VP exposure, potentially altering participants’ response patterns due to changes in context and experience. However, these variations did not have a significant impact on the domains assessed, indicating consistency and correlation among the items. In light of these findings, when interpreting the results of these specific items, attention should be paid to the timing of the application of the scale and the conditions that may make certain items more susceptible to temporal variation.

Four factors were found to be consistent with those in the original English version of the VPIRS. These factors successfully categorized the scored items. This result indicates that the VPIRS-E accurately assesses the same constructs as its original counterpart. Moreover, the VPIRS-E scale showed strong overall reliability with a Cronbach’s alpha of 0.82, closely matching the original scale’s value of 0.86 [18]. VPIRS-E also exhibited enhanced reliability in the fourth domain, achieving a score of 0.705, surpassing the original’s 0.662 [18]. Furthermore, no statistically significant differences were found among the various student groups, with a Cronbach’s alpha of 0.942 reported for the first domain, 0.751 for the second, 0.711 for the third, and 0.662 for the fourth. Based on the principal component’s analysis in the factor analysis modeling, item elimination was deemed unnecessary. Since the VPIRS-E was found to have consistent validity and reproducibility among students at various levels of undergraduate medical education, this scale can be broadly used in medical schools without being limited to a specific academic year. Our participants’ responses indicate that they had a favorable perception of the use of VPs. This result is consistent with those obtained in other studies, demonstrating the consistency and cross-contextual validity of the VPIRS in various educational and cultural contexts [24–27]. These results also suggest that the use of VPs in medical education is satisfactory for students [12, 28–30].

The congruence of the VPIRS-E with the original scale has implications for future research. By ensuring that measures of students’ perception of VP use are comparable across English and Spanish-speaking populations, comparative international studies on the role of VPs in undergraduate medical education can be conducted. Comparability allows for evaluating the impact of VPs in different educational contexts and cultures, potentially leading to enhanced best practices for integrating VPs into the curriculum and improving the learning experience with VPs as a complement to real clinical experiences. Areas of research that could benefit from this comparability include clinical skills development, the influence of VPs on self-learning, self-assessment, and online learning.

While the VPIRS-E has demonstrated reliability in assessing Spanish-speaking medical students’ perceptions, acknowledging the limitations of this study is crucial. The quantitative methodology employed provides robust measures of students’ perceptions, yet it may not fully capture the deep nature of their learning experiences with VPs. Qualitative data could illuminate the reasons behind our findings, such as students’ sense of motivation or potential dissatisfaction, and offer insights into their subjective learning experiences, like the perceived realism of the VPs or their influence on clinical reasoning skills. These descriptive insights could complement our findings by explaining the “why” behind the “what,” thereby enhancing our understanding of the effective integration of VPs into medical education.

The VPIRS-E scale is developed for use in diverse educational settings with the aim of assessing not only students’ perceptions of the learning process using virtual patients, but also other aspects of their integration into the curriculum. These aspects are mainly evaluated through dimensions 2 (items 15 to 18) and 4. Specifically, these dimensions explore students’ perceptions of the time required for the instructional strategy, the role of facilitators (whether present or absent), and the opportunities provided for fostering autonomy in learning. Under the pedagogical model proposed for our program’s students, it was necessary to use a scale that assumed a much more independent student-tool interaction that did not require the constant supervision of professors/facilitators during the process. Scales such as the one proposed by Huwendiek and colleagues integrate several individual aspects of the clinical reasoning process into a single item. Considering the importance of independently evaluating how the virtual patient differentially impactsthe development of skills such as clinical history taking, physical examination, and differential diagnosis, we believe it is necessary that each item can be evaluated separately.

Based on our experience, we recommend considering specific factors of the curriculum and educational settings that may influence students’ learning processes in the analysis of VPIRS-E results. This will help obtain a contextual understanding that addresses the needs of each institution and student population. We recommend presenting scale items in descending order to mitigate the primacy effect [31]. Similarly, when administering the scale, students should be given an average of 15 min to complete the scale, and a facilitator should be present to answer any questions students may have as they review the items.

## Conclusions

The reliability and validity of VPIRS-E across different student levels and its cultural adaptability highlight its significance for global medical education research. The VPIRS-E enables a more comprehensive and inclusive understanding of virtual patient integration in medical curricula by bridging linguistic and cultural gaps.

## Electronic supplementary material

Below is the link to the electronic supplementary material.

Supplementary Material 1

## Data Availability

The datasets used and/or analyzed during the current study are available from the corresponding author on reasonable request.

## Abbreviations

AVE: Average variance extracted
KMO: Kaiser-Meyer-Olkin
PBL: Problem-based learning
SD: Standard deviation
VPs: Virtual patients
VPIRS: Virtual patient integration rating scale
VPIRS-E: Virtual patient integration rating scale-español
