# Supplementary material for: Transcultural adaptation of the virtual patient integration rating scale: a factor analysis
Source: BMC Med Educ. 2025 Mar 18;25:396. doi: 10.1186/s12909-024-06571-z (PMC11921690; doi:10.1186/s12909-024-06571-z)
Supplement: Supplementary file 1 — Supplementary Material 1 [file 12909_2024_6571_MOESM1_ESM.docx]

**Supplementary material**

**Versión final VPIRS-E**

**Nombre del participante:**

**Semestre actual que cursa:**

**Instrucciones**

El siguiente cuestionario pretende evaluar el nivel de satisfacción con relación a uso de pacientes virtuales. Es muy importante que seas sincero(a) en tus respuestas y que sepas que no hay respuestas correctas o incorrectas pues nuestro objetivo es conocer cómo ha sido tu experiencia.

El tiempo estimado para resolver el cuestionario es de 7 a 8 minutos.

En relación a cada enunciado debes responder seleccionando una opción entre 1 a 5, siendo 1 “total desacuerdo” y 5 “totalmente de acuerdo”.

Por ejemplo: “Me gusta el café”

Marcas 1 si estás totalmente en desacuerdo, 2 si estás en desacuerdo, 3 si no estás en acuerdo ni en desacuerdo, 4 si estás de acuerdo y 5 si estás totalmente de acuerdo.

Si persiste o se presenta alguna duda o inquietud de los enunciados indícale a tu instructor.

**Insertar pregunta ejemplo**

“Disfruto de comer helado”

**Escala de Percepción de la Integración de Pacientes Virtuales al Currículo**

**Dimensión 1: Adquirir y retener el conocimiento**

1. Aprender a través de pacientes virtuales me ha ofrecido mucho conocimiento nuevo.
2. Los pacientes virtuales me han ofrecido la oportunidad de profundizar en el conocimiento que he adquirido hasta ahora
3. Los diferentes métodos de enseñanza que se han utilizado con los pacientes virtuales (fotos, texto) han hecho que aprender sea más fácil.
4. Aprender con pacientes virtuales me ha facilitado una mejor comprensión del cuidado integral del paciente.
5. El examen físico de los pacientes virtuales me permite retener más fácil el conocimiento
6. El requisito de proponer un diagnóstico diferencial me ayudará a obtener la habilidad de razonamiento clínico y priorizar el diagnóstico más probable cuando encuentre a un paciente con síntomas similares en la sala de espera.
7. Tengo un mejor conocimiento de cómo tratar pacientes con síntomas similares a los estudiados en los pacientes virtuales.
8. He aprendido más acerca de las presentaciones clínicas de las enfermedades relevantes para el trabajo práctico.
9. Los casos de los pacientes virtuales son un componente curricular apropiado
10. He aprendido más sobre el abordaje general del paciente mediante los pacientes virtuales.
11. El programa de pacientes virtuales me ha ayudado a desarrollar habilidades para una anamnesis dirigida.
12. El programa de pacientes virtuales me ha ayudado a desarrollar habilidades para un examen físico dirigido.
13. Los pacientes virtuales me han ayudado a desarrollar mi pensamiento para ampliar los diagnósticos diferenciales.
14. He aprendido cómo actuar con pacientes en escenarios específicos.

**Dimensión 2 Gestión del aprendizaje**

1. Las otras obligaciones académicas dejan tiempo suficiente para mi estudio independiente de los pacientes virtuales.
2. Las diferentes obligaciones académicas relacionadas con el paciente virtual se pueden llevar a cabo al mismo tiempo. (Obligaciones académicas entendidas como: construcción de historia clínica, revisión de literatura en torno al caso, preparación de la presentación del caso e identificación de hallazgos claves.)
3. Es una ventaja tener la oportunidad de decidir por mi cuenta cuándo completar mis tareas de estudio con mi paciente virtual.
4. Los pacientes virtuales son una herramienta útil para la educación médica continuada. (Entiéndase educación médica continuada como actividades diferentes a los cursos obligatorios)

**Dimensión 3 Enseñanza inauténtica.** (Enseñanza auténtica entendida como la estrategia que conecta a las asignaturas con el mundo real)

1. La educación con pacientes virtuales no es el aprendizaje teórico auténtico.
2. Tengo dudas de que el tratamiento del paciente virtual se corresponda con el del paciente real
3. En muchas ocasiones, los casos de pacientes virtuales son demasiado ideales.

**Dimensión 4: Desventajas para el Aprendizaje**

1. Los casos fueron muy exigentes para mi nivel de conocimiento.
2. Los casos de los pacientes virtuales ocuparon demasiado de mi tiempo.
3. No poder discutir los casos de pacientes virtuales con mi profesor era una desventaja.
4. Entendí poco los casos de los pacientes virtuales en inglés
